# A pragmatic and ethical guide for addressing life‐sustaining treatments in patients with suicidal thoughts or behaviors

**DOI:** 10.1002/jhm.70106

**Published:** 2025-06-17

**Authors:** Nurlan Aliyev, Chad Vokoun, Lou A. Lukas

**Affiliations:** ^1^ University of Nebraska Medical Center Omaha Nebraska USA; ^2^ Nebraska‐Western Iowa, Veterans Health Administration Omaha Nebraska USA

## INTRODUCTION

Over 12 million adults have serious suicidal thoughts each year, with nearly 2 million acting on those thoughts. Suicidal impulses are twice as common in people with chronic illness, so hospitalists commonly admit people with thoughts or actions of self‐harm. Those with clearly altered mental status receive full resuscitative efforts until their sensorium clears, but patients who are alert and oriented may also have significantly impaired decision‐making capacity. Making treatment decisions without accurately determining capacity risks the patient receiving treatment inconsistent with their values and puts clinicians at risk of providing treatment without proper consent. The article uses a common clinical situation to explore clinical practices, reviews ethical precepts, and offers a framework for clinical decision making in the face of questionable capacity.

## CASE PRESENTATION

*“Mr. Smith is a 32‐year‐old male with a history of depression who presents to ER with fever and shortness of breath. He was found to have lobar bacterial pneumonia with acute hypoxia. Hospital medicine was consulted for admission. Upon interview, Mr. Smith reveals that a week ago, he tried to “drink himself to death.” Several days later developed a cough, and when he had a hard time breathing, he got scared and came to the hospital, but adds, “Maybe I should have just stayed home and died.”*

A short hospitalization and mental health consultation is warranted, but the EMR won't process the admission without a Code Status order, which offers two options: “Full Code” and “DNR.”

## CLINICAL CONUNDRUM

This familiar situation can cause an ethical and pragmatic dilemma for clinicians. If asked directly about code status, the patient may refuse CPR, but a reasonable clinician may be concerned that this response may be colored by temporary despair and not reflect his true values; DNR request of a suicidal patient remains a topic of debate among experts.^1^, ^2^, ^3^, ^4^, ^5^ The patient may lack decision‐making capacity and require a surrogate decision maker, but it is unclear whether a clinician should violate a stable, communicative person's privacy by contacting a surrogate. Finally, some clinicians may be tempted to select the full code option without a conversation with the patient, rationalizing this decision either as “full treatment while stabilizing a patient” or that given this young healthy patient's risk of cardiac arrest, a discussion of code status is unwarranted. Finally, the clinician might find it ironic that the EMR requires a response for the unlikely event of cardiac arrest, but it does not prompt decisions more worthy of conversation, given his infection, such as respiratory failure or hypotension.

## SUICIDE AND DECISION MAKING

According to the 2021 National Survey of Drug Use and Health reports, 12.3 million adults aged 18 or older reported having serious suicidal thoughts, and 1.7 million adults attempted suicide in the United States.^6^ Alcohol use disorders and depression are the most prevalent comorbidities of suicidal patients, affecting 40%–60% respectively.^7^ People with chronic illness are twice as likely to experience suicide as the general population.^8^

Despite the self‐destructive and seemingly irrational nature of a suicide attempt, suicidality alone does not preclude future rational decision‐making. Suicide attempts are often impulsive, time‐limited acts that do not influence underlying cognition and judgment. After the impulse has passed, patients may retain the ability to make insightful, proactive health‐related decisions.^3^ Like all seriously ill patients, they require individualized assessment to assure they have sufficient capacity to make the medical decisions at hand.

## ETHICAL PRINCIPLES AND CLINICAL DECISION MAKING

Healthcare professionals balance foundational bioethical principles, including autonomy, beneficence, and non‐maleficence, in clinical practice.^9^ Respecting autonomy, which includes informed consent, truth telling, and maintaining privacy, balances power between clinicians and patients. The clinician formulates medical assessments and recommendations, while the patient has the power to accept or refuse those recommendations. Autonomy depends on the patient's ability to make rational decisions, and it is that ability that must be assessed and documented in cases of attempted or idealized suicide.

Applebaum provides a structured approach to determining a patient's ability to make decisions that has long guided medical‐legal practice. He offers a sequence of increasing complicated cognitive tasks that substantiate the ability to make decisions that are easily assessed in the conduct of a normal medical interview: (1) communicating a choice; (2) understanding relevant information; (3) appreciating the situation and its potential medical consequences; and (4) reasoning through the medical recommendations.^10^

## CASE APPLICATION

In this case, Mr. Smith is able to hear and speak well enough that his ability to communicate a choice is not in doubt. He is able to discuss the data you present about his pneumonia, so the second criteria is also met. He seems to understand that the infection is serious enough to warrant hospitalization and antibiotics, but he denies having an alcohol problem and says he is not depressed. However, his affect is flat, and he feels hopeless and helpless about his future, and makes ongoing references to going to sleep and not waking up. When you mention that occasionally people in his condition require aggressive life‐saving treatment, he says that his life isn't worth saving.

At this point, a prudent clinician should determine that he lacks the capacity to make decisions that impact his survival because he is rationalizing decisions to forgo treatment based on feeling worthless, a symptom of acute depression. Contacting a surrogate may feel like it detracts from autonomy, but the opposite is true because it assures that there is someone to advocate on behalf of the patient and balance the role of the clinician who is making recommendations. Further, contacting a surrogate decision maker protects the clinician should the patient be harmed by a decision that was made when the patient may have lacked capacity.

In this situation of what appears to be temporary incapacity, a surrogate will usually approve treatment that preserves life, but obtaining the surrogate's input does not assure that aggressive life‐sustaining treatment would be accepted. For example, the surrogate may provide additional unexpected information‐ previously undisclosed serious illness, uncommon religious beliefs, or any factor that would reveal a genuine and long‐term value consistent with not accepting certain medical treatments. At this point, the clinician is reminded that beneficence (doing good) doesn't always mean preserving life; it means providing treatment that is consistent with the patient's goals and values.

If the surrogate does not know the patient's values, they are advised to follow substituted judgment by inferring what the patient would have wanted based on other things known about them, and finally, what is in the patient's best interests.^11^ In complex cases, assistance from social work or an ethics consultant can be helpful. Additionally, early involvement of mental health professionals, especially in cases involving suicidal ideation, is essential. Psychiatric evaluations can help distinguish between transient suicidal thoughts and more enduring cognitive impairments, thereby supporting accurate assessments of decision‐making capacity. Integrating mental health input into the initial evaluation process ensures that both medical and psychological dimensions of care are addressed.

## SYSTEMIC ISSUES

An unexpected finding from this review was that this clinical conundrum of dealing with code status on admission may be the iatrogenic effect of hospital policy reflected in electronic order sets rather than legislative or regulatory requirements. Though it is widely believed that the Patient Self Determination Act^12^ drives these policies, our review of the literature refutes this and, in fact, found examples of alternate processes, including the Veterans Administration's Life Sustaining Treatment Decisions initiative that only requires clinician initiation of discussion of life sustaining treatment for high‐risk individuals.^13^

To address these challenges, we propose that EMR systems be designed to allow deferral of code status entry when decision‐making capacity is uncertain or a psychiatry evaluation is pending. Hospitals could implement clinician‐driven prompts rather than mandatory fields for code status and include ethics consultation options when suicidality or questionable capacity is documented. These changes could reduce moral distress, support clinical judgment, and ensure a more ethical, patient‐centered approach.

**Figure 1:**
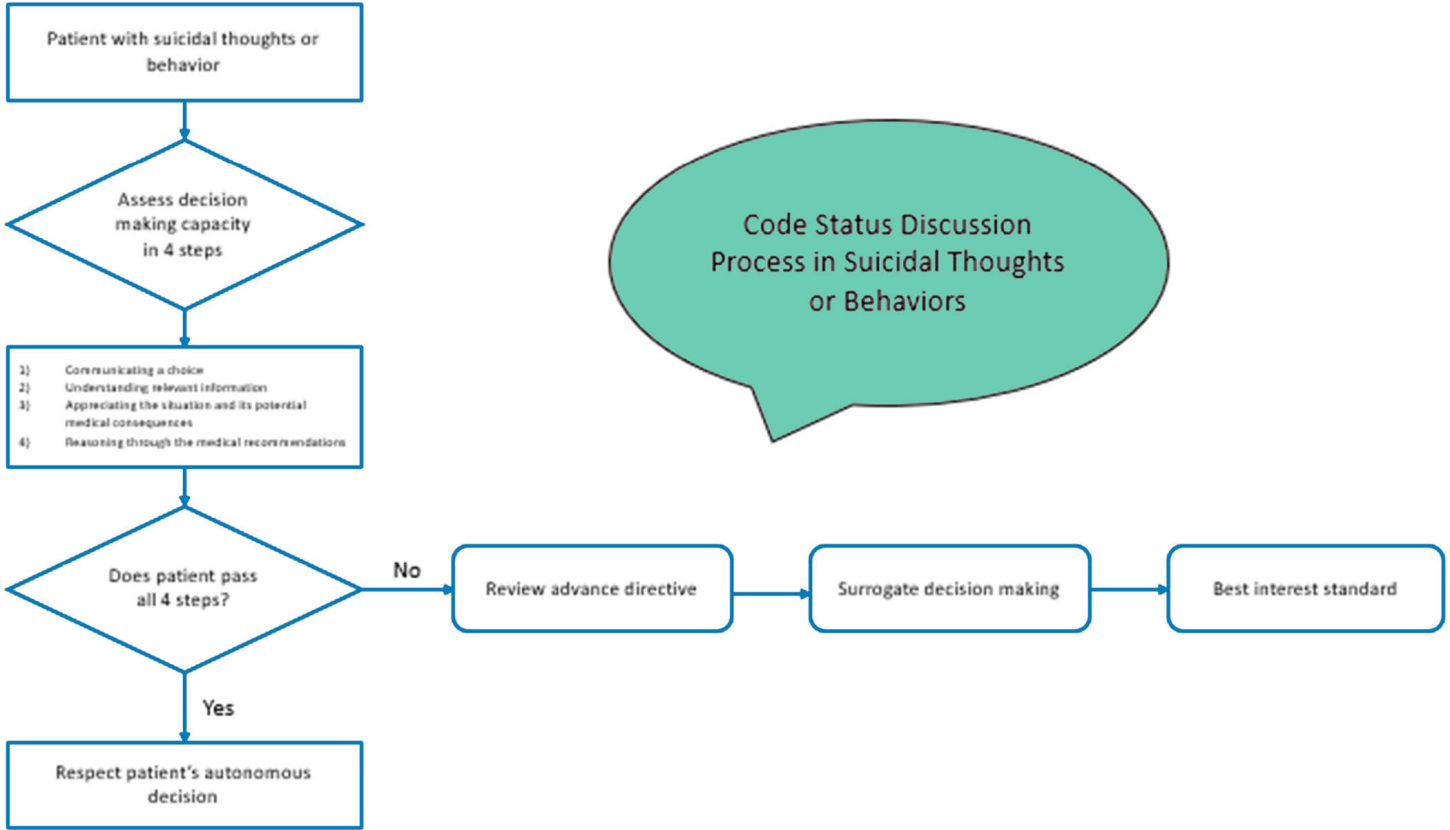
Algorithm for guiding code status discussions in patients with suicidal thoughts or behaviors.

In conclusion, the raw number of people who have serious thoughts of suicide or suicidal actions and the increased prevalence of these intentions among people with chronic illness means that hospitalists routinely admit and make treatment plans for patients with comorbid suicidal ideation or actions. Clinicians may doubt these patients' ability to make sound medical decisions, especially regarding life‐sustaining treatments, but self‐harming impulses, even in people with other mental illnesses or substance use disorders, do not *automatically* invalidate a patient's ability to make rational decisions. To ensure ethical treatment and protect against liability, prudent clinicians should carefully screen all patients for decision‐making capacity using a structured approach before seeking decisions, code status, and life‐sustaining treatment (Figure 1). Systemic factors that force conversations that are not clinically indicated may have unintended effects.

We also recognize the practical barriers clinicians face in applying this framework. Time constraints, limited access to mental health consultants, and differences in training or comfort with ethical deliberation can all influence real‐world implementations. To mitigate these challenges, we suggest using brief validated capacity assessment tools, incorporating clinical decision support within EMRs. Simulation training and mentorship can further support clinicians facing ethically complex cases.

## SCOPE OF THE PAPER

Addressing code status with suicidal patient on inpatient admission. What to do aftermath of suicidal attempt who has previous DNR is an ethical dilemma and is beyond the scope of this article.

## CONFLICT OF INTEREST STATEMENT

The authors declare no conflicts of interest.
